# Circulating tumor cell viability during and after radiotherapy mirrors treatment response in cancer patients

**DOI:** 10.1002/1878-0261.70261

**Published:** 2026-04-23

**Authors:** Yvonne Goy, Jana Löptien, Cornelia Coith, Afroditi Nanou, Katharina Hintelmann, Pinnschmidt Hans, Cordula Petersen, Klaus Pantel, Sabine Riethdorf, Kerstin Borgmann, Harriet Wikman

**Affiliations:** ^1^ Department of Radiotherapy and Radiation Oncology University Medical Center Hamburg‐Eppendorf Hamburg Germany; ^2^ Department of Tumor Biology, University Medical Center Hamburg‐Eppendorf Hamburg Germany; ^3^ Department of Medical Cell BioPhysics, Faculty of Science and Technology University of Twente Enschede the Netherlands; ^4^ Mildred Scheel Cancer Career Center HaTriCS4 University Medical Center Hamburg‐Eppendorf Hamburg Germany; ^5^ Institute of Medical Biometry and Epidemiology University Medical Center Hamburg‐Eppendorf Hamburg Germany

**Keywords:** brain and bone metastases, breast cancer, CTC, lung cancer, radiotherapy

## Abstract

The response to radiotherapy (RT) is influenced by the individual DNA repair capacity of both the tumor cells and the host. In this study, we assessed whether circulating tumor cell (CTC) enumeration, kinetics, and CTC viability (i.e., the apoptotic rate) could provide a more accurate tool for monitoring and stratifying the RT response. We analyzed CTCs and tumor‐derived extracellular vesicles (tdEVs) during RT in 71 lung (*n* = 35) and breast (*n* = 36) cancer patients receiving RT treatment for brain (*n* = 54) and bone (*n* = 19) metastases. The DNA repair capacity of the host was assessed by *ex vivo* irradiation of peripheral blood mononuclear cells (PBMCs). RT treatment did not seem, in most cases, to cause a short‐term release of CTCs. We found that both the number and apoptotic rate of CTCs before and after RT treatment is a powerful indicator of poor prognosis. Additionally, the fraction of apoptotic CTCs correlated with RT response and patient outcome. This study demonstrated that the RT response is associated with tumor‐specific traits, which can be accessed via easily accessible liquid biopsy approaches.

## Graphical abstract

Radiotherapy (RT) response depends on the DNA repair capacity of tumor and host cells. We show that circulating tumor cell (CTC) counts and apoptosis rates before and after RT predict treatment response and outcome, which can be accessed via easily accessible liquid biopsy approaches. Created in BioRender. Wikman, H. (2026), https://BioRender.com/we1vuhe.

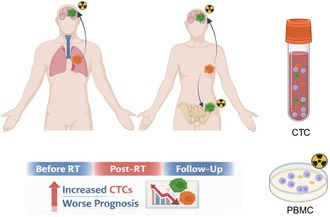

**Abbreviations**
BoM: bone metastasis
BrM: brain metastases
CT: computed tomography
CTC: circulating tumor cell
FUP: follow‐up
Gy: Gray
MRI: magnetic resonance imaging
NSCLC: nonsmall cell lung cancer
OS: overall survival
PBMC: peripheral blood mononuclear cells
RT: radiotherapy
SFRT: stereotactic fractioned radiotherapy
SRS: single‐fraction radiosurgery
tdEV: tumor‐derived extracellular vesicles

## 1. Introduction

Radiotherapy (RT) is a fundamental component of standard therapy for cancer patients, including those in the metastatic setting. Currently, radiation oncologists have limited tools for predicting or assessing treatment response beyond medical follow‐up imaging. However, the assessment of the tumor response through imaging is only possible with a temporal delay, and subclinical changes remain hidden.

Furthermore, with multiple systemic therapies being incorporated into cancer treatment, critical questions arise regarding the optimal scheduling of RT to effectively synergize with these new therapy options [1, 2]. RT not only induces DNA damage and cell death in tumor cells but also triggers abscopal effects, that is, systemic responses through DNA damage and systemic immune signaling [3, 4]. The potential risks of metastasis arising from the complex changes induced by radiation in the tumor microenvironment have long been debated, as they may undermine the long‐term effectiveness of treatment [5]. For example, radiation‐induced endothelial/vascular injury affects endothelial permeability by disrupting tight and adherence junctions [6], potentially fostering the release or mobilization of tumor cells into the bloodstream [5].

Circulating tumor cells (CTCs), the seeds of metastasis, can be detected with high sensitivity in both early‐ and late‐stage cancer patients [7]. CTCs represent an easily accessible source of tumor cells that are associated with patient outcomes. Baseline CTC counts and changes over the course of treatment have been shown to reflect the response to therapy. However, simply enumerating CTCs is insufficient to understand the mechanisms driving metastasis; therefore, a detailed characterization of tumor cells is essential [8]. The presence of apoptotic CTCs is already common in untreated patients [9, 10, 11], with more apoptotic CTCs in metastatic patients than early‐stage patients [12].

RT causes extensive DNA damage in tumor cells, and the ability of each cell to repair this damage plays a significant role in the radioresponse [13]. The repair capacity is defined by both inherited variations and somatic mutations in DNA repair genes commonly found in tumors [14]. Thus, monitoring and characterizing CTC viability, that is, the apoptotic rate and kinetics, could provide a more accurate tool for monitoring and stratifying the RT response. Tracking CTC counts and characteristics during RT could predict treatment response or resistance, leading to recurrence.

In this study, we enumerated and characterized CTCs during the course of RT in nonsmall cell lung cancer (NSCLC) and breast cancer patients treated for brain and bone metastases, along with the DNA damage response profiles of normal cells. The aim of this study was to evaluate whether CTCs are released upon RT treatment and whether CTC counts and apoptosis, together with the host response, could be used as prognostic and predictive markers for individualized RT response in patients with breast cancer and NSCLC.

## 2. Materials and methods

### Patients

All 71 patients included in this study were treated at the Department of Radiotherapy and Radiation Oncology at University Hospital Hamburg. Blood was collected from NSCLC and breast cancer patients undergoing RT of bone or brain metastases at three different time points (between January 2017 and October 2019) (Tables 1 and 2). All (*n* = 35) NSCLC patients were treated for brain metastases whereas 19 breast cancer patients received RT for brain and 19 for bone metastases (two patients RT treated for both brain and bone metastases). In two additional breast cancer patients, blood was taken within two different series of RT of consecutively occurring brain metastases.

**Table 1:** Study population and clinical data.

		All BrM	NSCLC BrM	Breast ca. BrM	All BoM
Patients^a^	All	54	35	19	19
Sex	Female	42	23	19	19
	Male	12	12	0	0
Age at PD (y)	Mean	58.3	62.8	50.5	58.1
	Range	34–77	44–77	34–67	35–88
Age at BrM‐RT (y)	Mean	62.1	64.5	57.7	65.3
	Range	44–84	44–84	46–71	40–90
Primary^b^ metastasized	Yes^a^	10	4	6	7
	No	40	28	12	11
Oligo‐BrM^c^	Yes	19	18	1	
	No	26	9	17	
Subtype breast^d^ ca.	Luminal			4	7
	Basal			5	2
	HER2			4	5
No. of BrM^e^	1	15	11	4	
	2–10	18	13	5	
	> 10	13	5	8	
Type of RT^f^	SRS	11	8	3	0
	SFRT	43	27	16	15
Survival^g^	Alive	18	14	4	10
	Dead	30	18	12	9
FUP (month)	Range	0.5–81.6	0.5–62	0.5–81.6	0.5–36.2
	Mean	13.1	13.4	12.3	17.6

Abbreviations: BrM: brain metastases, BoM: bone metastasis, PD: primary diagnosis, RT: radiotherapy, F: female, M: male, No: number, FUP: follow‐up, Y: year, SRS: single‐fraction radiosurgery, SFRT: stereotactic fractioned radiotherapy.

^a^ Two patient was diagnosed and treated for both brain and bone metastases.

^b^ Missing info for four BrM patients and one BoM patient.

^c^ Missing info for five BrM patients.

^d^ Missing info for one BrM and one BoM.

^e^ Missing info for 8 BrM patients.

^f^ Missing info for four BoM patients.

^g^ Missing info for six BrM patients.

**Table 2:** CTC positivity rates.

		Baseline			Post‐RT			MRI/CT		
		*n*	Pos.	%	*n*	Pos.	%	*n*	Pos.	%
All samples										
≥ 2 CTC		75	26	34.7	54	18	33.3	22	3	13.6
≥ 5 CTC		75	19	25.3	54	11	20.4	22	3	13.6
Brain metastases										
≥ 2 CTC	All	56	15	26.8	41	12	29.3	17	2	11.8
	NSCLC	35	6	17.1	25	5	25.0	11	2	18.2
	Breast^a^	21	9	42.9	16	7	43.7	6	0	0
≥ 5 CTC	All	56	11	19.6	41	7	17.1	17	2	9.1
	NSCLC	35	4	11.4	25	3	12.0	11	2	18.2
	Breast^a^	21	7	33.3	16	4	25.0	6	0	0
Bone metastases										
≥ 5 CTC	Breast	19	8	42.1	13	4	30.8	5	1	20.0

^a^ Including two patients with RT treatment for two different brain metastases with negative CellSearch results and two patients with RT treatment for both brain and bone metastases.

The first blood draw was taken just before RT treatment (baseline, *n* = 71 patients with 75 samples). In 50 patients (54 samples), blood was also collected within 30 min after the final dose of RT (post‐RT), and in 20 patients (22 samples), blood was taken 6–14 weeks after the last dose at the time of magnetic resonance imaging (MRI for brain metastases) or CT (bone metastases) (follow‐up group). Fifty‐four patients (*n* = 56 samples) were treated with RT for brain metastases. Thirty‐five of the brain metastases patients had NSCLC, and 19 had breast cancer as the primary tumor type. Nineteen female patients received RT for bone metastases. The mean follow‐up time (FUP) was 13.1 months. All bone‐metastatic patients received stereotactic fractioned RT (SFRT, total 30–54 Gray (Gy), 5–27 fractions), whereas 43 of the brain metastatic patients received SFRT (total 25–39 Gy, 5–10 fractions) and 11 received single‐fraction radiosurgery (SRS, 23–24 Gy) (Table 1).

All participants provided written informed consent prior to blood donation, which was approved by the local ethical committee (No. PV‐5392, 06/12/2016, Ärztekammer Hamburg). The study methodologies conformed to the standards set by the Declaration of Helsinki. In accordance with the approval, collection of clinical samples and laboratory experiments were separated and performed by different persons blinded to clinical diagnosis and outcomes.

### CTC analyses

CellSearch analyses were performed on 7.5 mL of EDTA blood, which was processed within 24 h of blood collection as previously described [15]. CTCs were enriched with EpCAM antibodies and detected by the application of antibodies against keratins (CTC marker) and CD45 (negative marker to exclude leukocytes). DAPI was used to stain the nuclear material. Cell morphology (keratin staining pattern, nuclear size, and morphology) was assessed to define the apoptotic state of CTCs as previously described [16]. In a subset of samples, M30 (M30 CytoDeath, VLVbio, Nacka, Sweden), an additional apoptosis marker, was added to the 4th channel of the CellSearch system.

Two different CTC cutoffs were used depending on the primary tumor origin. For breast cancer patients, the FDA‐approved cutoff of ≥ 5 CTCs/7.5 mL of blood was used. For NSCLC patients as well as for the whole study cohort, both ≥ 2 and ≥ 5 CTCs/7.5 mL of blood were used on the basis of pooled data results on NSCLC [17].

### Tumor‐derived extracellular vesicles (tdEV) analyses

For tdEV enumeration, the digitally stored fluorescence image files were processed with open‐source ACCEPT software v1.1 [18] via the ‘Full Detection’ function. After image processing, a linear gate for enumerating tdEVs was applied as described before [19, 20]. The definition of tdEV was EpCAM+, CK+, DAPI−, and CD45− particles with a diameter less than 14 μm. More specifically, the tdEV gates were as follows: mean intensity marker 1 ≤ 5, maximum intensity CK > 90, size CK ≤ 150 μm^2^, perimeter CK > 5 pixels, eccentricity CK ≤ 0.8, and perimeter to area CK ≤ 1.

### Blood sample analyses

Blood samples were taken from 19 patients in heparinized tubes. Peripheral blood mononuclear cells (PBMCs) were separated from 10 mL whole‐blood samples by Ficoll‐Paque (Merck, Darmstadt, Germany) density gradient centrifugation at 600 × g for 45 min and washed in PBS within 24 h (Miltenyi Biotec, Bergisch Gladbach, Germany). Isolated PBMCs were incubated at 37 °C in RPMI‐1640 medium (Pan‐Biotech, Aidenbach, DE) supplemented with 10% fetal bovine serum (Gibco Life Technologies, Mulgrave, VIC, Australia). PBMCs were irradiated with 6 Gy (200 kV, 1.2 Gy/min, Gulmay) or sham irradiated and then further incubated for 24 h at 37 °C.

### Immunofluorescence staining of double‐strand breaks by γH2AX

The PBMC samples were cytospun onto microscope slides (Tharmac, Limburg an der Lahn, Germany), via a Cytospin 4 cytocentrifuge (Thermo Fisher‐Scientific, Waltham, MA, USA) for 4 min at 800 × g. The cell spots were dried for 10 min and outlined with a PAP pen (ProSciTech, Kirwan, QLD, Australia). PMBCs were permeabilized, fixed with 4% paraformaldehyde, and blocked overnight in 3% BSA. Foci were detected via primary antibodies against γH2AX [Ser139] (1 : 250, Millipore, Merck, Darmstadt, Germany) followed by the secondary antibody Alexa Fluor 488‐conjugated goat anti‐rabbit IgG (1 : 600, Cell Signaling Technology, Danvers, MA, USA). Nuclei were stained with DAPI, and samples were mounted (Vector Laboratories, Newark, CA, USA). γH2AX foci were quantified automatically via the Aklides® system (MediPan, Blankenfelde‐Mahlow, Germany). A minimum of 100 cells per dose and slide were quantified.

### Statistical analyses

Statistical analyses were performed using SPSS version 30 (IBM, USA). Correlations among ordinal or continuous data were assessed by Spearman correlation analyses. To assess differences between independent samples, chi‐square and Fisher's exact tests were employed for nominally scaled and dichotomous data, while Kruskal–Wallis and Mann–Whitney U‐tests were employed for continuous and ordinal data. For continuous and ordinal data of dependent samples, Friedman and Wilcoxon signed rank tests were used to assess differences. The fixed effects of RT‐time point, tumor location and time point‐by‐location interaction on CTC positivity were analyzed with a mixed effects logistic model estimating random intercepts for individual patients. Poisson regression was used to estimate CTC counts at post‐RT, based on ln(x + 1)‐transformed baseline CTC counts. An observed CTC > 50% over the model‐estimated value was considered an increase, whereas a CTC < 50% of the model‐estimated values was defined as decrease. Kaplan–Meier estimates with the log‐rank tests were used to analyze survival differences between the groups. A significance level of 0.05 was assumed to determine statistical significance. Curve fitting graphs and related statistics were generated via graphpad prism (version 6.02) software. The data are presented as the means (+SEMs) of at least three replicate experiments. When comparing results from the different time points, we first ascertained via chi square or Mann–Whitney U‐test, as appropriate, that drop out or missingness in follow‐ups was not associated with baseline characteristics.

## 3. Results

### CTC baseline enumeration

For the 71 patients included in this study, 155 CellSearch analyses were performed. Pre‐RT (baseline) CTC results were obtained from 75 samples, including four patients with two different and consecutively occurring metastases.

**Fig. 1:**
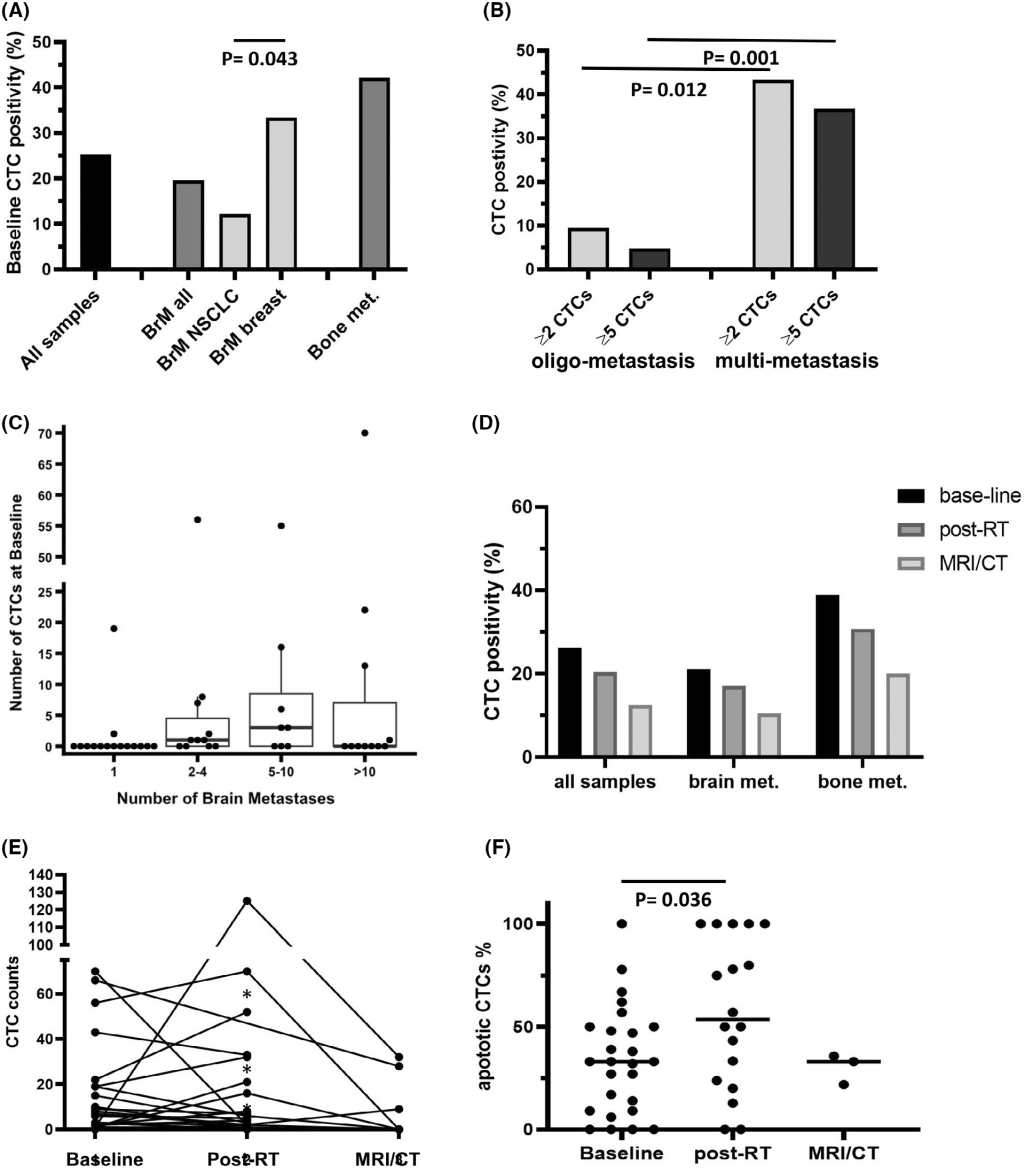
CTC enumeration. (A) Bar chart showing CTC positivity (% of patients having ≥ 5 CTC) rates in baseline samples (*n* = 75) divided by tumor entity and localization of RT therapy. Among brain metastatic patients (BrM), CTC‐positive status was significantly associated with breast cancer (*P* = 0.043, Fisher's exact test). (B) Correlation between CTC positivity (≥ 2 and ≥ 5 CTC) and oligo‐ *vs*. multi‐metastatic disease showed significantly less CTC‐positive cases among the oligo‐metastatic cases (*P* = 0.001, Fisher's exact test). (C) The number of brain metastases and number of CTCs are not associated with each other (*P* = 0.067, Kruskal–Wallis test). Error bar indicates 1.5‐fold of interquartile range. (D) CTC positivity (≥ 5 CTC) at different time points and in different patient cohorts, showing no significant differences between the time points (mixed effects logistic model). Dots show single patients' results; horizontal line indicates median. (E) Connected dot plot showing changes in CTC numbers in individual patients detected at baseline, post‐RT and at follow‐up MRI/CT. Three patients receiving SRS are marked with an *. (F) Percentages of apoptotic CTCs detected at different time points, showing a significant increase in post‐RT samples (*P* = 0.036, paired Wilcoxon rank sum test, horizontal line indicates median value of all samples).

Figure 1A and Table 2 show the CTC positivity rates in the different patient groups at baseline. Here, in 34.7% (26/75) of all patients, ≥ 2 CTCs/7.5 mL blood were detected, and in 25.3% (19/75) of patients, ≥5 CTCs/7.5 mL could be detected. Most CTCs were detected in breast cancer patients with bone metastases (≥ 5 CTCs, 8/19 42.1%), but the number of CTCs was not significantly greater than that in breast cancer brain metastases patients (≥ 5 CTCs 33.3%, *P* = 0.80, Fisher's exact test). Breast cancer patients with bone metastases had median CTC counts of three (mean 8.2, range 0–66). Breast cancer patients with brain metastases had a median CTC count of one (mean 14.5, range 0–112), and NSCLC patients with brain metastases had a median CTC count of one (mean 12.4, range 0–347) (data not shown). NSCLC patients with brain metastases had significantly fewer CTCs than did breast cancer patients with brain metastases (≥ 2 CTCs, *P* = 0.033 and ≥ 5 CTCs, *P* = 0.043, Fisher's exact test). Importantly, fewer CTCs were identified in patients with oligo‐brain metastatic disease (brain as only affected organ) than in patients with additional extracranial metastases (≥ 2 CTCs 2/21 vs 13/30, *P* = 0.012 and ≥ 5 CTCs 1/21 vs 11/30, *P* = 0.001, Fisher's exact test, Fig. 1B). No correlation between age, sex, Karnofsky index or type of RT treatment and CTC count was observed (data not shown). As the oligo‐brain metastatic state was significantly associated with fewer CTCs, we also assessed whether the number of brain metastases was associated with CTC counts (Fig. 1C). Whereas patients with one solitary brain metastasis had significantly less CTCs (2/15, mean 1.4 CTCs) compared to the rest (16/30, mean 9.1 CTCs; *P* = 0.016, Mann–Whitney U‐test), no correlation between the number of brain metastases and CTC counts was observed over all groups (*P* = 0.067, Kruskal–Wallis test).

### CTC enumeration during and after RT treatment

In 54 samples, post‐RT samples were available, and in 22 samples, CTC results were obtained at the time of MRI/CT (6 to 14 weeks after the last dose) (Table 2). A small but nonsignificant reduction in the positivity rates at post‐RT was seen for all groups as indicated by the mixed effects logistic model (Table 2 and Fig. 1D). The CTC positivity rates for the MRI were even lower, with only three CTC‐positive patients (two with brain and one with bone metastases), indicating successful RT treatment in most patients. No significance was, however, seen, due to low sample size (Table 2 and Fig. 1D).

Among the 22 samples from whom blood was collected at the different time points and whose CTCs were detected (≥ 2 CTC) at any of the points, a mixed response was observed (Fig. 1E). As the CTC values between baseline and post‐RT highly correlated, we assessed a possible increase or decrease in CTC status between time points using negative binomial regression analyses to predict the post‐RT CTC status. A > 50% deviation of the observed CTC counts from the model‐estimated counts was defined as increase respectively decrease. Whereas in 11.1% of patients an increase in CTC numbers was observed at post‐RT, in 14.8% a decrease in CTC numbers was observed. Interestingly, two of the three patients (66.6%) who received single‐fraction radiosurgery (STS) had increased CTC counts directly after RT (marked with asterisk in Fig. 1E), whereas among patients who received fraction RT, only 21.7% (4/23) had increased CTC counts.

### Apoptotic CTCs

Figures 1F and S1 show the average number of apoptotic CTCs identified in patients with ≥ 2 CTCs. Compared with those at baseline, significantly more apoptotic CTCs were detected in samples taken directly after the last RT (*P* = 0.036, paired Wilcoxon rank sum test). At baseline, the mean apoptotic rate of CTCs in patients was 34.7%, whereas after RT, 55.9% of detected CTCs were apoptotic. The apoptotic rate was increased very similarly among the different patient groups, with slightly more apoptotic CTCs in patients receiving RT for brain metastases (60.3%) than in those receiving RT for bone metastases (51.5%) (data not shown). At MRI/CT, only three patients had CTCs. In these cases, the number of apoptotic rate decreased (31.7%) and corresponded to those at baseline.

**Fig. 2:**
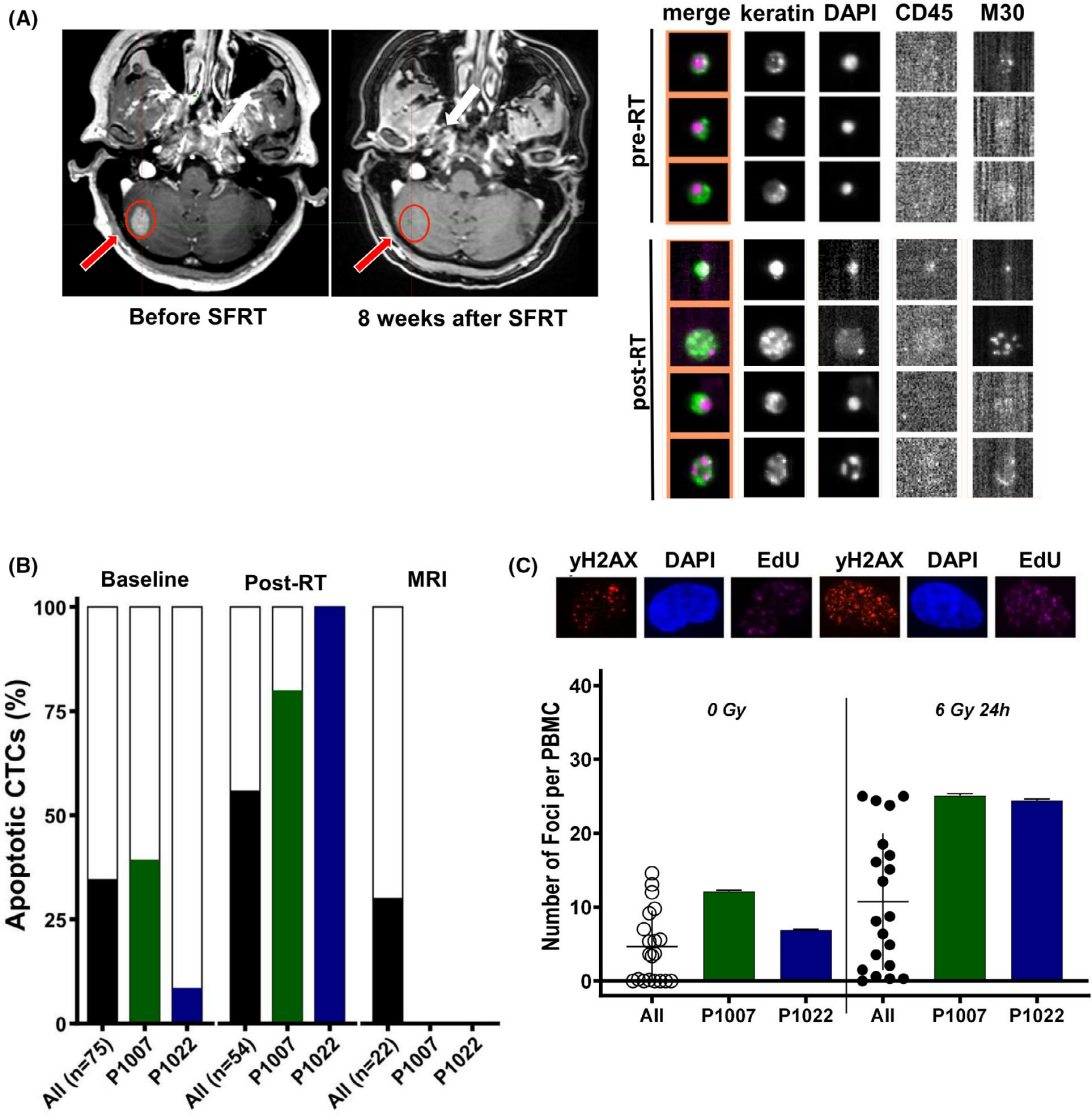
Correlation between RT response and the number and viability of CTCs and PBMCs. (A) Left: Brain MRI (magnetic resonance imaging) images before and after SFRT (stereotactic fractioned radiotherapy) treatment (10 × 3.0 Gy) of a brain metastasis from a female *BRCA1*‐germline mutant positive breast cancer patient (P1007) with complete remission after 8 weeks. Right: Exemplary images of non‐apoptotic and apoptotic CTCs pre‐RT and post‐RT, respectively. The patient had high apoptotic rates of CTC counts at baseline (22/56 CTCs) and even more apoptotic CTCs after RT (56/70 CTCs), with no signs of CTCs or metastases after 8 weeks. (B) A Bar plot showing both the mean percentages of apoptotic CTCs detected at different time points in all patients as well as in patients P1007 and P1022. (C) DNA damage in the PBMCs of 19 patients compared with patients P1007 and P1022 was analyzed by γH2AX staining. Nuclei were counterstained with DAPI, and foci were quantified via the MediPan device before RT (0 Gy) or 24 h after irradiation *in vitro* (6 Gy for 24 h). Data represented as mean ± SD.

Patients with regressive disease (*n* = 6) had, on average, 49.1% apoptotic CTCs (range 32%–67%), whereas 7 patients with progressive or mixed response had a lower percentage of apoptotic CTCs (33.8%, range 0%–100%) (*P* = 0.33, paired Wilcoxon rank sum test). Figure 2A,B and S2 show examples of two brain metastatic patients in whom both the CTC numbers and the number of apoptotic cells correlated closely with the RT response.

### PBMC analyses

**Fig. 3:**
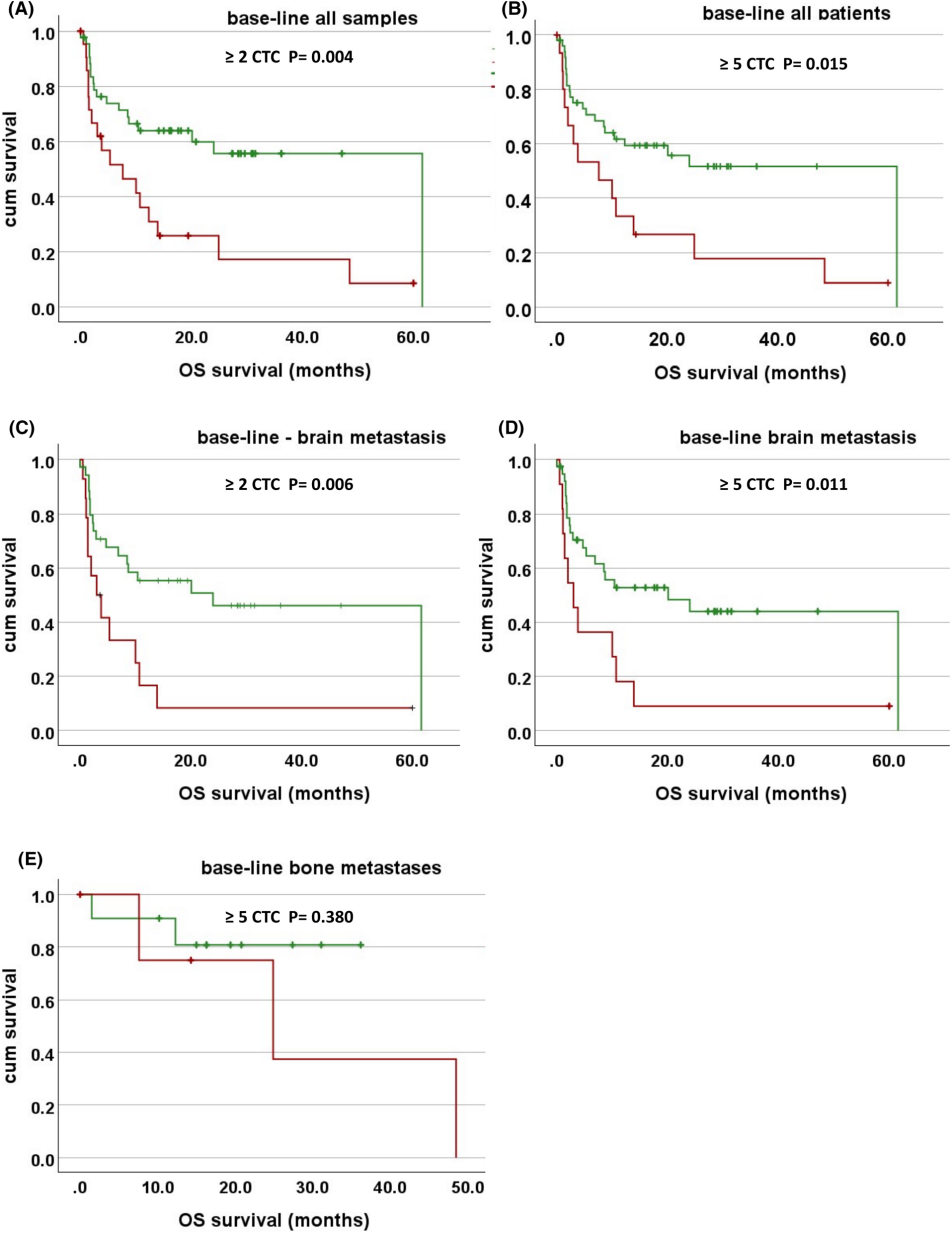
Kaplan–Meier survival curves (log‐rank test) for overall survival (OS) stratified by baseline CTC levels. The OS of all patients (*n* = 69) significantly decreased when either two (A) or five (B) CTCs/7.5 mL of blood were detected (red line. *P* = 0.004 and *P* = 0.015, respectively) compared to CTC negative patients (green line). Patients with brain metastases (*n* = 50) have a significantly decreased OS when positive for either ≥ 2 or ≥ 5 CTCs (*P* = 0.006 and *P* = 0.011) (C and D), whereas CTC numbers in bone‐metastatic patients (*n* = 19) are not associated with OS in baseline samples (*P* = 0.380) (E).

In 19 of the 75 patients examined, the DNA damage response in PBMCs was analyzed in parallel with CTCs [21]. The analysis of DNA damage visualized by γH2AX staining revealed a broad distribution ranging from 0 to 14.59 foci/per cell, with a mean value of 4.7+/−1.1 SEM foci/cell. The percentages of apoptotic cells or γH2AX foci before RT (Pre), at the end of RT (post) and at the time of MRI/CT were not significantly different (Fig. 3A,B). Interestingly, after *ex vivo* irradiation of the PBMCs, the two patients who achieved complete remission after RT (Fig. 2B) also presented a strong increase in DNA damage, as measured by the formation of γH2AX foci (Fig. 2C).

### Survival analyses

During the time of the study, 36 patients died (54.5%). The median follow‐up after the first blood draw was 14.7 months (range: 0.5–61.6). When all the baseline samples were analyzed together, both ≥ 2 and ≥ 5 CTCs/7.5 mL of blood were significantly associated with shorter overall survival (OS, *P* = 0.004 and *P* = 0.015, respectively) (Fig. 3A,B). Both ≥ 2 and ≥ 5 CTCs were significantly associated with OS in all patients with brain metastases (*P* = 0.006 and *P* = 0.011, respectively, Fig. 3C,D); but a ≥ 5 cutoff was not significant for patients with bone metastases (*P* = 0.308) (Fig. 3E). When breast cancer patients and NSCLC patients with brain metastases were analyzed separately, a significant association with poorer outcome was observed only in the breast cancer group (Fig. S4A–S4C).

**Fig. 4:**
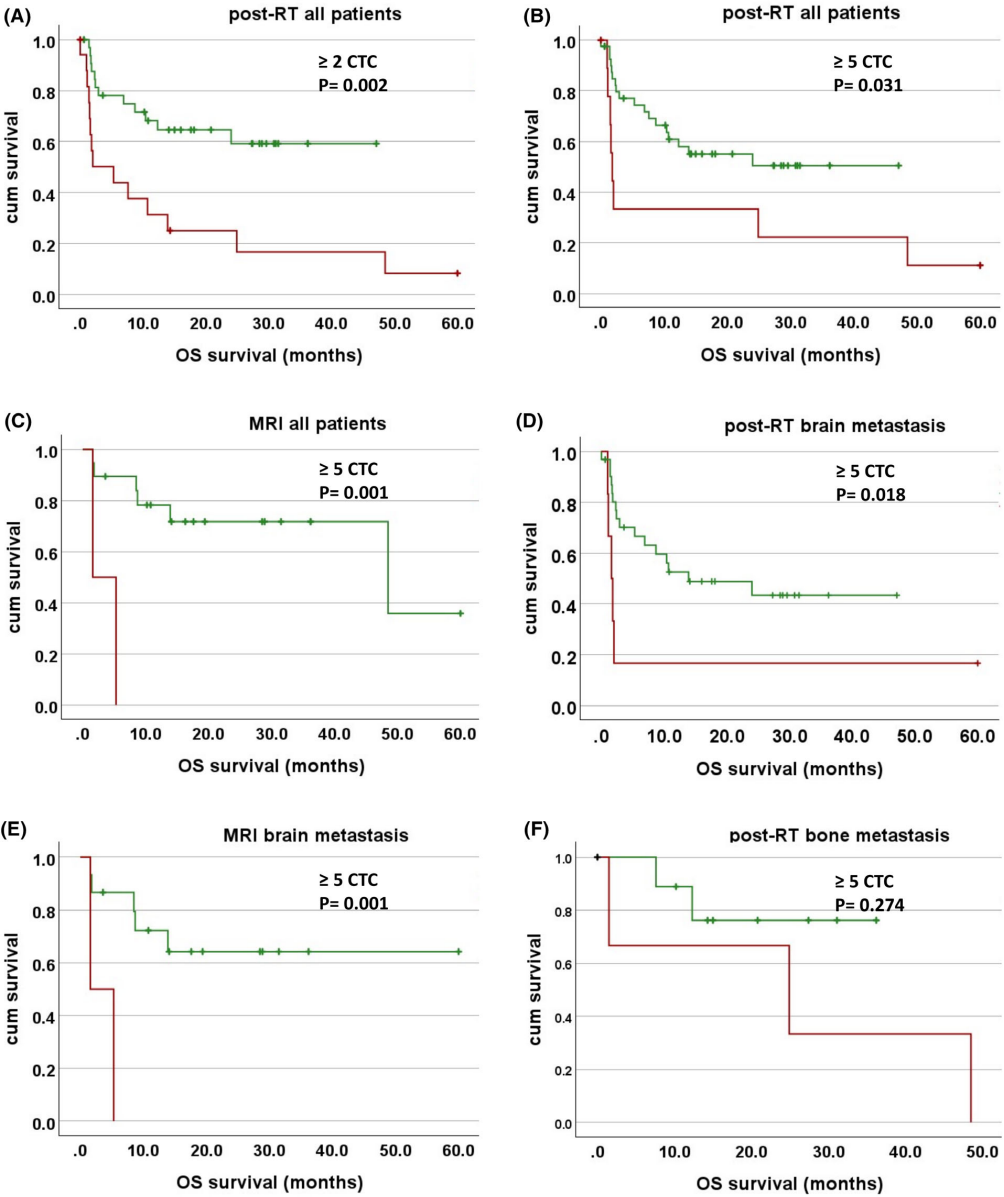
Survival analyses based on CTC levels detected post‐RT and at the time of MRI/CT. (A,B) At post‐RT, the OS of all patients (*n* = 51) significantly decreased when either two (*P* = 0.002) or five CTCs/7.5 mL of blood (red line) (*P* = 0.031) were detected (Kaplan–Meier log‐rank test). (C) At MRI, all patients (*n* = 21) showed a significantly decreased OS when positive for ≥ 5 CTCs (*P* = 0.001). (D,E) ≥ 5 CTCs detected at either post‐RT (*n* = 38) or MRI/CT (*n* = 17) revealed a significant association with poor prognosis in brain metastatic patients (*P* = 0.001 and *P* = 0.018, respectively), whereas CTC numbers in bone‐metastatic patients are not associated with OS at post‐RT (*n* = 13) (F).

When all post‐RT samples were analyzed, both ≥ 2 and≥ 5 CTC cutoffs were significantly associated with OS (*P* = 0.002 and *P* = 0.031, Fig. 4A,B). This was also observed for samples taken at the time of MRI/CT (≥ 5 CTCs/7.5 mL, *P* = 0.001, Fig. 4C). Similar to baseline samples, ≥ 5 CTCs at either post‐RT or MRI/CT were significantly associated with all brain metastases (post‐RT *P* = 0.018; follow‐up MRI *P* = 0.001, Fig. 4D,E) and especially with breast cancer brain metastases but not for NSCLC (post‐RT *P* < 0.001, Fig. S4D,S4E). For the small cohort of breast cancer bone metastases patients, again no significance was seen at post‐RT (Fig. 4F). For NSCLC brain metastases, follow‐up at MRI samples with ≥ 5 CTCs was associated with poor survival (*P* = 0.048, Fig. S4F). These data indicate that a positive diagnosis for CTCs is strongly associated with poor overall survival.

**Fig. 5:**
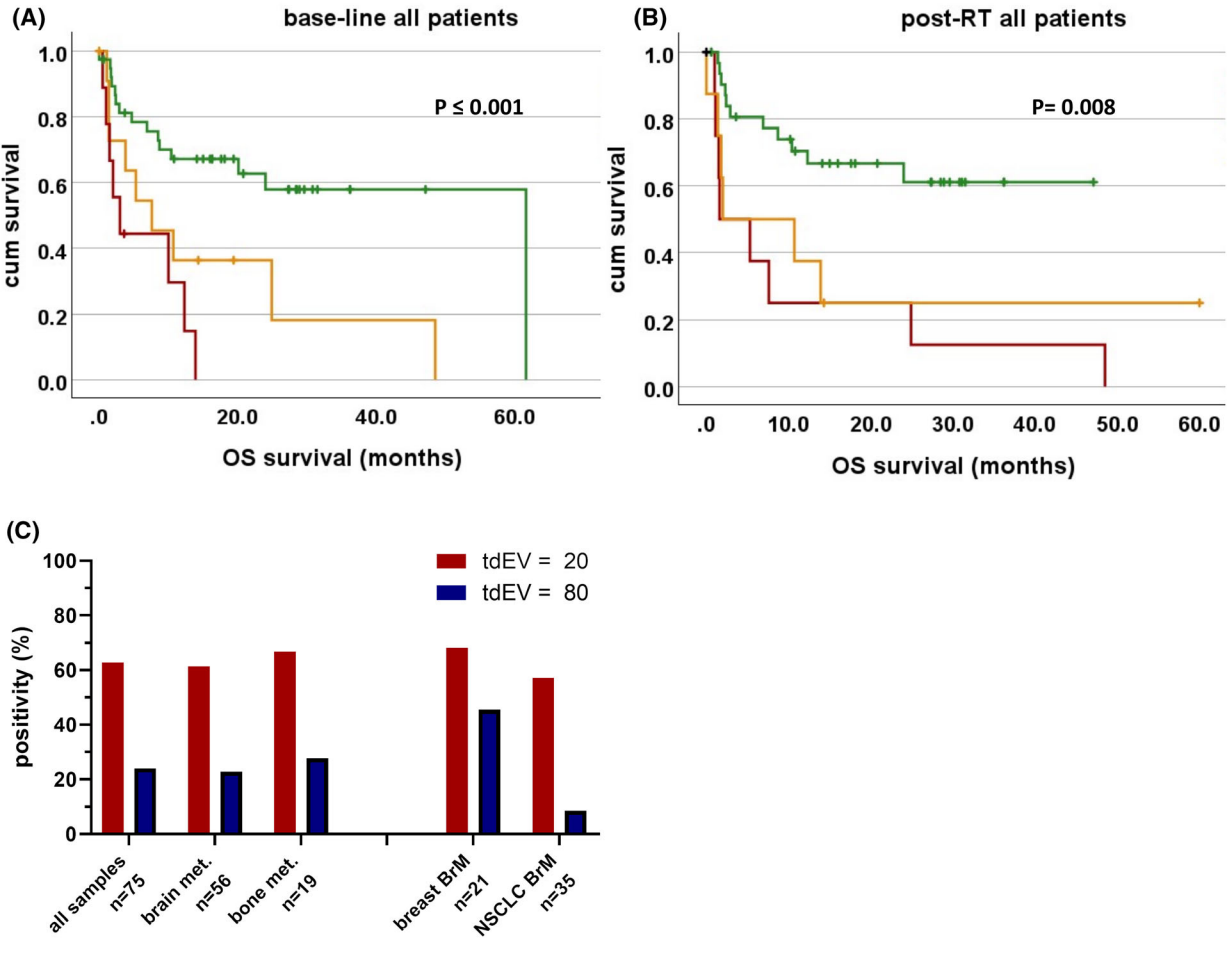
CTC stratification by the apoptotic rate and tdEV detection. Kaplan–Meier curves (log‐rank test) for baseline (*n* = 60) (A) and post‐RT (*n* = 50) (B) samples stratified for no (green line), intact (red line), or apoptotic CTCs (orange line), with the mean percentage of apoptotic CTCs used as the cutoff. Patients with intact CTCs showed a significant reduced survival compared to apoptotic or no CTCs, with patients that harbor no CTCs have the best survival outcome (*P* < 0.001 and *P* = 0.008). (C) tdEV positivity rates detected in different patient cohorts.

When we separated the CTC‐positive patients on the basis of their mean percentage (34.7%) of apoptotic CTCs, a clear survival benefit was observed for patients at baseline and post‐RT, with more apoptotic CTCs than for those with more intact CTCs (*P* < 0.001 and *P* = 0.008, pooled over strata) (Fig. 5A,B).

### tdEV analyses

tdEV amounts were also assessed for the baseline samples. The median number of tdEVs was 31.0 tdEVs/7.5 mL of blood (range 0–2659), with no significant differences between the different patient groups. The median tdEV number was, however, similar to the CTC data, greater among the breast cancer patients (48.5 and 58.0 for bone and brain metastases, respectively) than among those in the NSCLC brain metastasis group (27.0, Fig. 5C). Two different cutoffs (≥ 20 and ≥ 80/7.5 mL blood) for defining tdEV positivity have been described in the literature [19, 20]. Although the absolute tdEV number correlated significantly with the CTC count (*P* = 0.001), it alone was not associated with survival in any of the patient groups (data not shown). When patients were stratified as having either tdEVs (≥ 80) or CTCs (≥ 5), a significant association with positive status was found for the whole study population (*P* = 0.022), but with less discrimination power compared to CTC analyses alone. A significant association was also seen for all brain metastases (*P* = 0.038) and especially for breast cancer brain metastasis patients (*P* = 0.01), with a median survival of only 7.2 months, whereas the median survival time was 21.7 months for patients who were negative for both markers. For breast cancer brain metastases patients, adding the tdEV data slightly increased the median survival time compared to when only the CTC status were considered (8.8 versus 14.7 months for CTC‐positive versus negative patients) (Fig. S5A–S5C).

## 4. Discussion

Different types of liquid biopsies have been shown to provide powerful real‐time monitoring tools for the sequential tracking of various tumor‐derived molecules and cells found in peripheral blood, thus providing a substitute for tissue biopsies [7]. Numerous earlier studies have demonstrated that the progression‐free survival (PFS) and overall survival (OS) of cancer patients are correlated with the presence of CTCs [22]. In this study, we enumerated CTCs and assessed their apoptotic phenotype at different time points during RT treatment. We found that both the number and apoptotic rate of CTCs before and after RT treatment are powerful prognostic indicators. Furthermore, RT treatment does not seem, in most cases, to cause the release of CTCs associated with a worse prognosis.

Similar to our previous findings, we detected significantly more CTCs among breast cancer patients than among NSCLC patients [23]. Furthermore, as in earlier results [24], NSCLC patients with brain metastases seem to have very low levels of EpCAM‐positive CTCs, as only 17.1% of NSCLC brain metastatic patients had > 2 CTCs/7.5 mL of blood. Interestingly, although the oligo‐metastatic (defined here as only brain) disease setting, indicative of a lower tumor burden, was associated with fewer CTCs, this was not observed when it was correlated with the absolute number of brain metastases. These findings suggest that the blood–brain barrier may be more efficient at impeding the release of CTCs into the peripheral circulation than extracranial metastases are. Nevertheless, these CTCs are clinically relevant and associated with prognosis, especially when they are found post‐treatment.

In an animal model, it was shown that, immediately after high‐dose RT, CTC numbers increase but only for 20 min before returning to pretherapy levels [25]. In our study, this was not observed in patients treated with fractionated lower‐dose RT. Interestingly, 2 out of 3 patients with positive CTC status and receiving a single high‐dose RT had a short‐term increase in CTCs. Importantly, all patients with increased CTC counts post‐RT and/or MRI/CT compared with baseline CTCs died, with a mean survival of only 2.7 months, compared with 16.7 months among those with stable or decreased CTC numbers. Furthermore, irrespective of the time point of CTC analysis, CTC positivity was significantly associated with shorter OS. The correlation with poor prognosis was especially strong among breast cancer brain metastases patients, with samples taken either post‐RT or via MRI/CT. A clear limitation of these analyses is the low number of samples analyzed at the later time points.

We also used previously published open‐source image analysis software for the detection of tdEVs in baseline samples. tdEVs are defined as particles between 1 and 14 μm in size that coexpress epithelial cell adhesion molecules and cytokeratin but not leukocyte‐specific CD45 and show no DNA stain and are associated with shorter OS in metastatic cancers of epithelial origin [19, 20, 26]. Although tdEV numbers correlated significantly with CTC counts, they were not associated with patient outcome. Although a combined CTC positivity and tdEV status was significantly associated with shorter OS especially in breast cancer brain metastasis patients, CTC analyses alone performed better.

Radioresistance and radiosensitivity vary greatly between tumors, depending on the cell type and the genetic makeup of the patient [27, 28]. Thus, we expected that CTCs originating from the tumor site receiving RT would show evidence of DNA damage in a patient‐specific manner, which is correlated with patient response. Although we did not find a significant increase in the total number of CTCs directly after RT, we detected significantly more apoptotic CTCs in samples taken directly after the last RT than in baseline samples. Interestingly, at the MRI follow‐up, the number of apoptotic CTCs decreased again in the few samples with CTCs and corresponded to baseline levels, indicating that RT treatment could cause a short‐term release of damaged CTCs. Clearly more samples would need to be analyzed for a definitive answer. In a recent paper, Niedermayer et al. also looked at CTC numbers and DNA damage response in breast cancer patients during chemo/targeted treatment. Similar to our results, the number of CTCs did not increase during the treatment but the DNA damage response as measured by γH2AX response was significantly increased [29]. Thus, as observed in the RT‐treated cohort and in other studies, persistence or an increase in both the number and the apoptotic rate of CTCs after treatment is a powerful indicator of poor prognosis [30, 31, 32]. In a large study by Deutsch et al. (2017), the authors evaluated the impact of apoptotic CTCs in many (*n* = 442) metastatic breast cancer patients via the CellSearch system [16]. Although no brain metastatic patients were included in the study, the authors reported that the kinetics of apoptotic CTCs had a stronger discriminating power than intact CTCs alone. They also reported a strong correlation between apoptotic and intact CTCs in baseline samples. Similarly, a study by Jansson et al. [11] revealed that the presence of apoptotic CTCs after therapy was associated with worse survival, indicating that the occurrence of apoptotic CTCs is both an intrinsic, patient‐dependent tumor trait and an important marker for treatment success [11].

## 5. Conclusion

In conclusion, our data indicate that the radioresistance could be assessed via different easily accessible liquid biopsy approaches. Furthermore, our pilot analyses indicated that host factors, assessed by *ex vivo* irradiation of PBMCs, could be additional useful parameters for assessing individual RT response, potentially in combination with other liquid biopsy biomarkers.

## Conflict of interest

The authors declare no conflicts of interest.

## Author contributions

YG, KB, and HW designed the study; YG, KH, CP, and KP recruited patients; YG, CC, SR, and AN performed the experiments; YG, JL, HP, AN, KB, and HW performed the statistical analysis and generated the figures and tables; YG and HW drafted the manuscript. All authors provided intellectual input and approved the manuscript.

## Supporting information


**Fig. S1.** Histogram showing both the total number of CTCs as well the number of apoptotic CTCs at different time points.
**Fig. S2.** Correlation between RT response and the number and viability of CTCs.
**Fig. S3.** Percentage of apoptotic PBMCs and number of γH2AX in 19 breast cancer patients under RT.
**Fig. S4.** Kaplan Meier curves for overall survival (OS) in different patient sub‐cohorts using both ≥ 2 and ≥ 5 CTC as cut off.
**Fig. S5.** Kaplan–Meier curves for patients with a combination of high tdEV and ≥ 5 CTCs.

## Data Availability

The data supporting the results of this study are available upon reasonable request from the corresponding authors.
