# Supplementary material for: Circulating tumor cell viability during and after radiotherapy mirrors treatment response in cancer patients
Source: Mol Oncol. 2026 Apr 23;20(8):2040–53. doi: 10.1002/1878-0261.70261 (PMC13398918; doi:10.1002/1878-0261.70261)
Supplement: Supplementary file 1 [file MOL2-20-2040-s001.zip › Supporting-Information-new.docx]

**Supporting Information**

**Supplementary Figure 1.** Histogram showing both the total number of CTCs as well the number of apoptotic CTCs at different time points.

**Supplemental Figure 2.** Correlation between RT response and the number and viability of CTCs. Brain MR images before and after SRS treatment (1 × 24.0 Gy.) of four brain metastases from a female NSCLC cancer patient, with no signs of metastases or CTCs after 10 weeks. Before the start of treatment, the patients had 56 CTCs and the patients survived 60 months.

**Supplementary Figure 3.** Percentage of apoptotic PBMCs and number of γH2AX in 19 breast cancer patients under RT. (A) No significant changes in apoptotic cells in PBMCs before (pre), at the end (post) and at the time of MRI (MRI), automatically counted by the Aklides® NUK system (Medipan) (Spearman’s correlation)). (B) No significant changes in DNA damage under RT calculated as the number of γH2AX in PBMCs of the same 19 patients. Data represented as mean ± SEM.

**Supplemental Figure 4.** Kaplan Meier curves (log rank test) for overall survival (OS) in different patient sub-cohorts using both ≥ 2 and ≥ 5 CTC as cut off. OS of NSCLC brain metastatic patients is not significantly decreased when either two (A) or five (B) CTCs/7.5ml blood were detected at baseline. Breast cancer patients with brain metastases have a significantly decreased OS (C). Similarly, at post-RT CTC numbers in NSCLC patients where not associated with OS whereas a significant association was found for the breast cancer cohort (E). ≥ 5 CTC in NSCLC brain metastatic patients at MRI was significantly associated with poor prognosis

**Supplemental Figure 5.** Kaplan–Meier curves (log rank test) for patients with a combination of high tdEV and ≥ 5 CTCs (solid line) showing significantly decreased OS for all patients (A), and for all brain metastatic (B) and breast cancer brain metastatic patients (C).
