# Supplementary material for: Circulating tumor cell viability during and after radiotherapy mirrors treatment response in cancer patients
Source: Mol Oncol. 2026 Apr 23:10.1002/1878-0261.70261. Online ahead of print. doi: 10.1002/1878-0261.70261 (PMC13398918; doi:10.1002/1878-0261.70261)
Supplement: Supplementary file 1 — Fig. S1. Histogram showing both the total number of CTCs as well the number of apoptotic CTCs at different time points. Fig. S2. Correlation between RT response and the number and viability of CTCs. Fig. S3. Percentage of apoptotic PBMCs and number of γH2AX in 19 breast cancer patients under RT. Fig. S4. Kaplan Meier curves for overall survival (OS) in different patient sub‐cohorts using both ≥ 2 and ≥ 5 CTC as cut off. Fig. S5. Kaplan–Meier curves for patients with a combination of high tdEV and ≥ 5 CTCs. [file MOL2-9999-0-s001.zip › Suppl_Figures.pptx]

## Slide 1
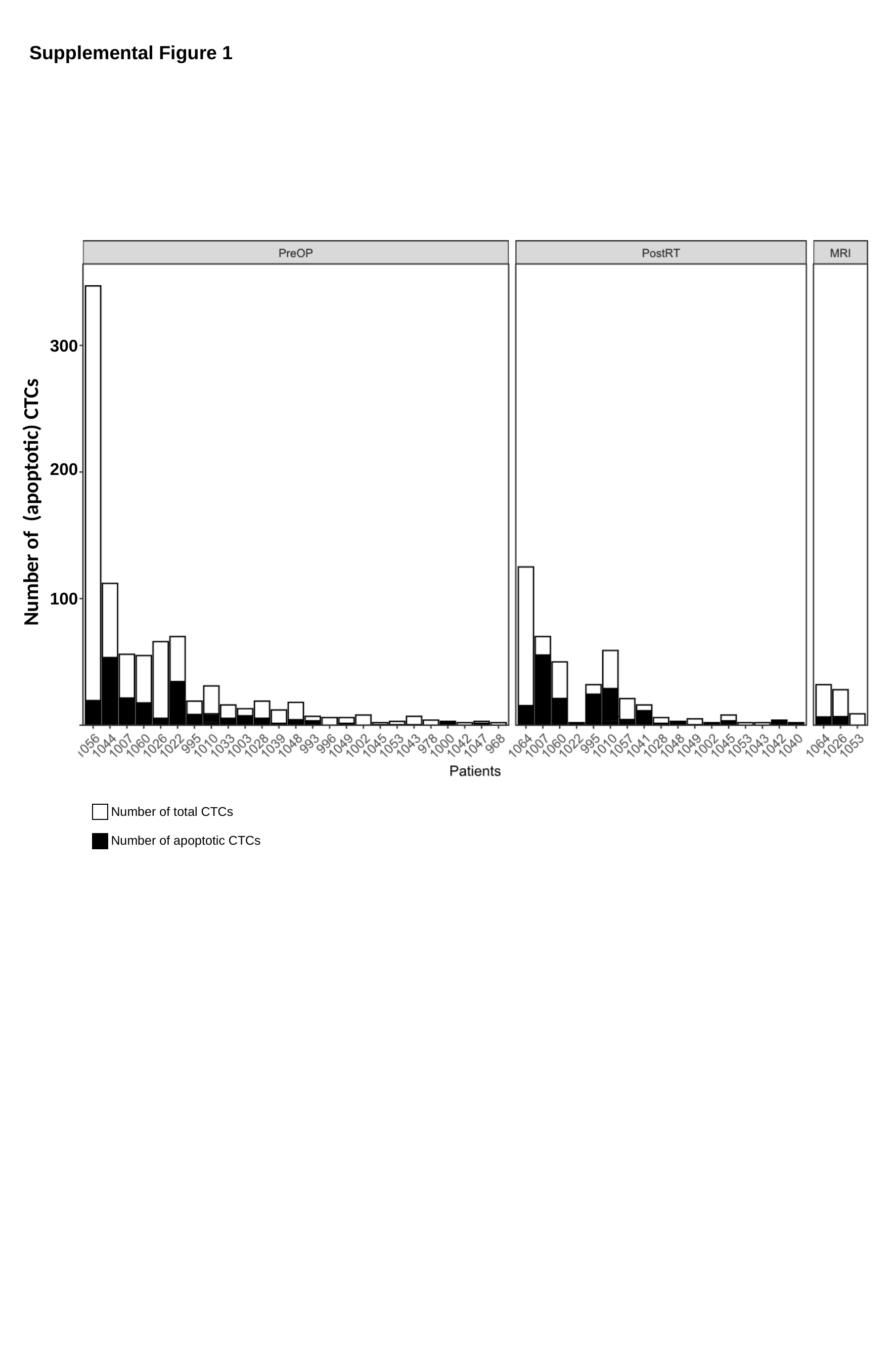

Supplemental Figure 1
300
200
Number of (apoptotic) CTCs
100
C
Number of total CTCs
Number of apoptotic CTCs

## Slide 2
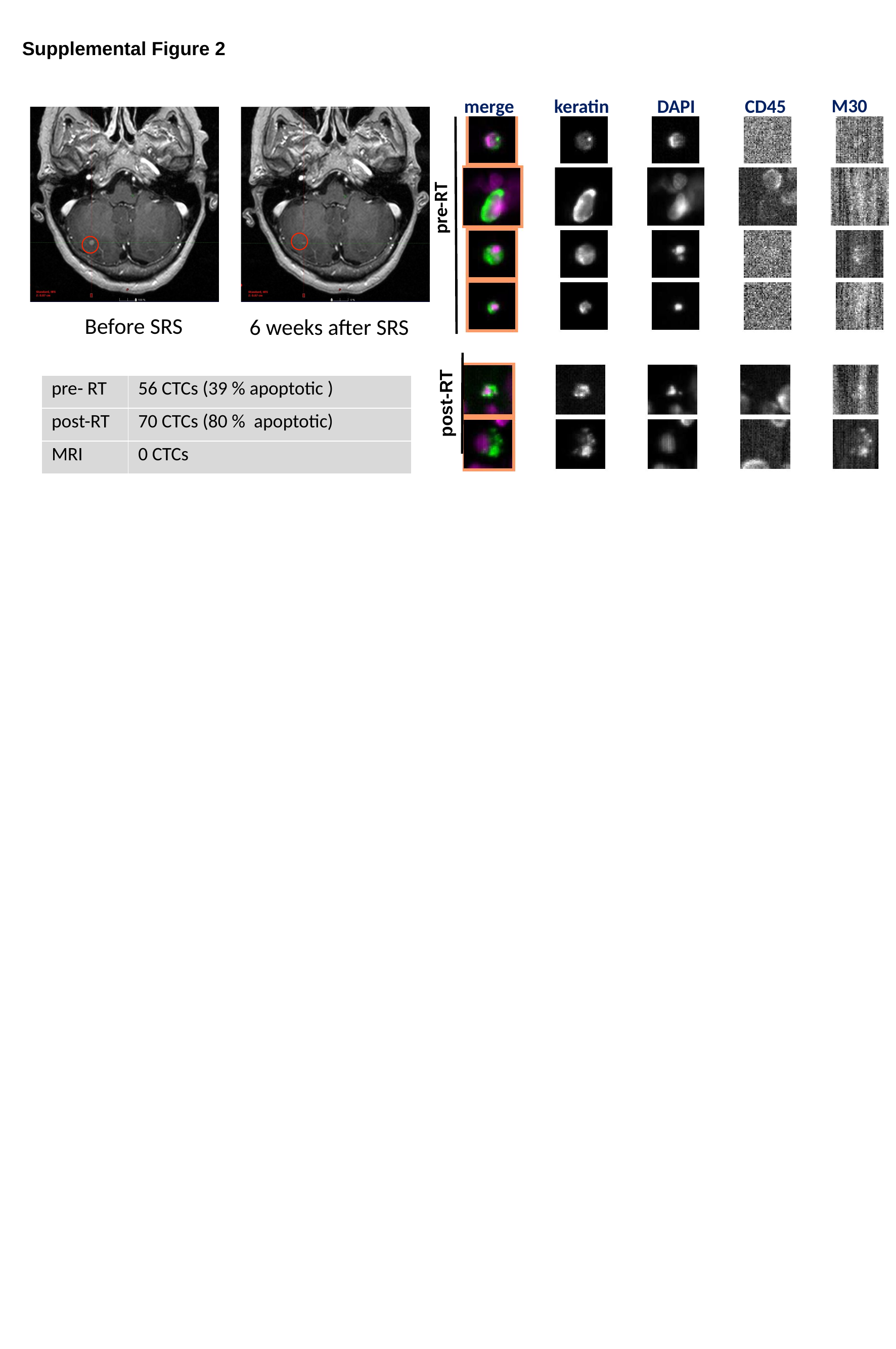

Supplemental Figure 2
M30
keratin
DAPI
CD45
merge
pre-RT
Before SRS
6 weeks after SRS
| pre- RT | 56 CTCs (39 % apoptotic ) |
| --- | --- |
| post-RT | 70 CTCs (80 % apoptotic) |
| MRI | 0 CTCs |
post-RT

## Slide 3
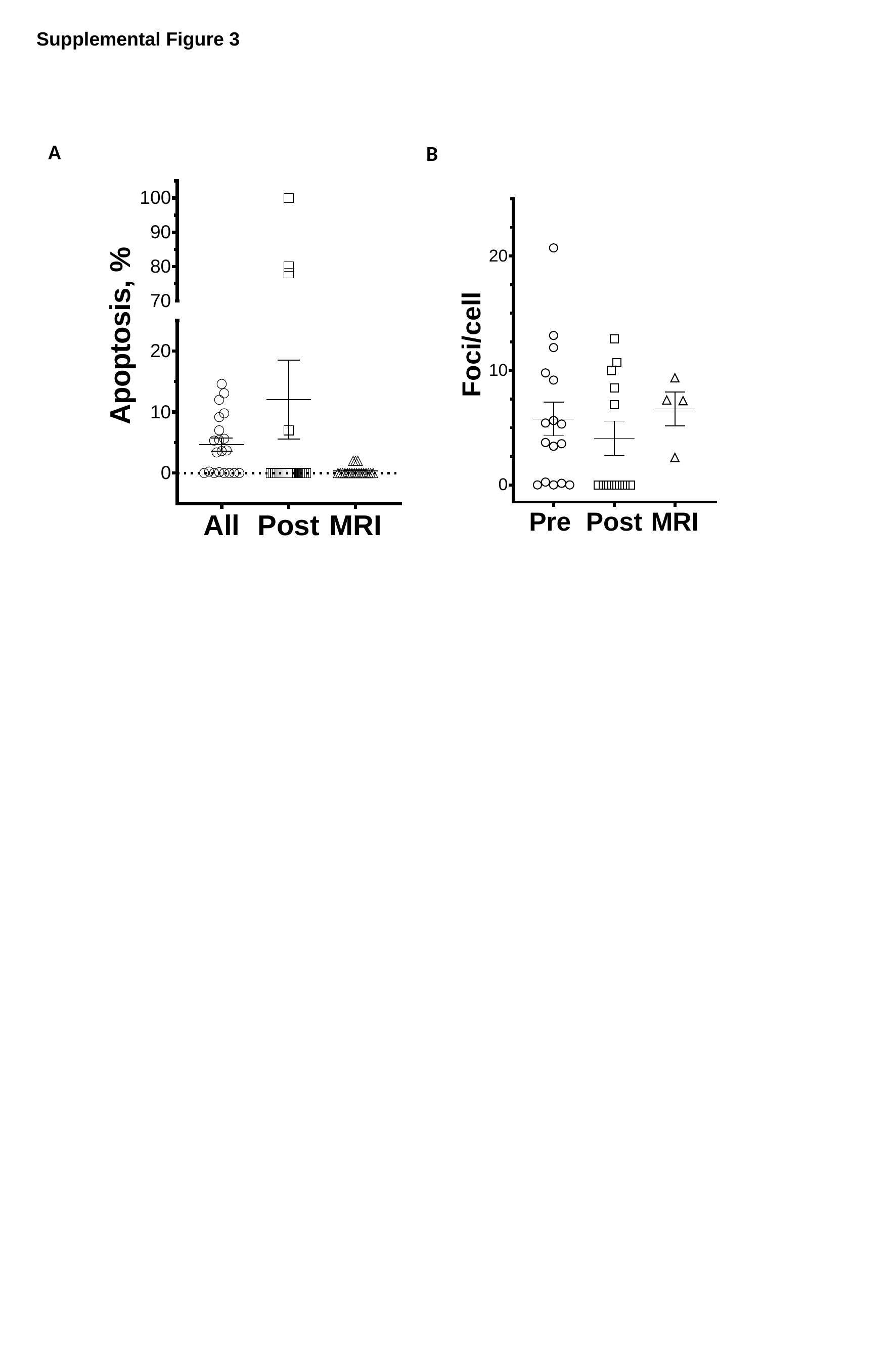

Supplemental Figure 3
A
B

## Slide 4
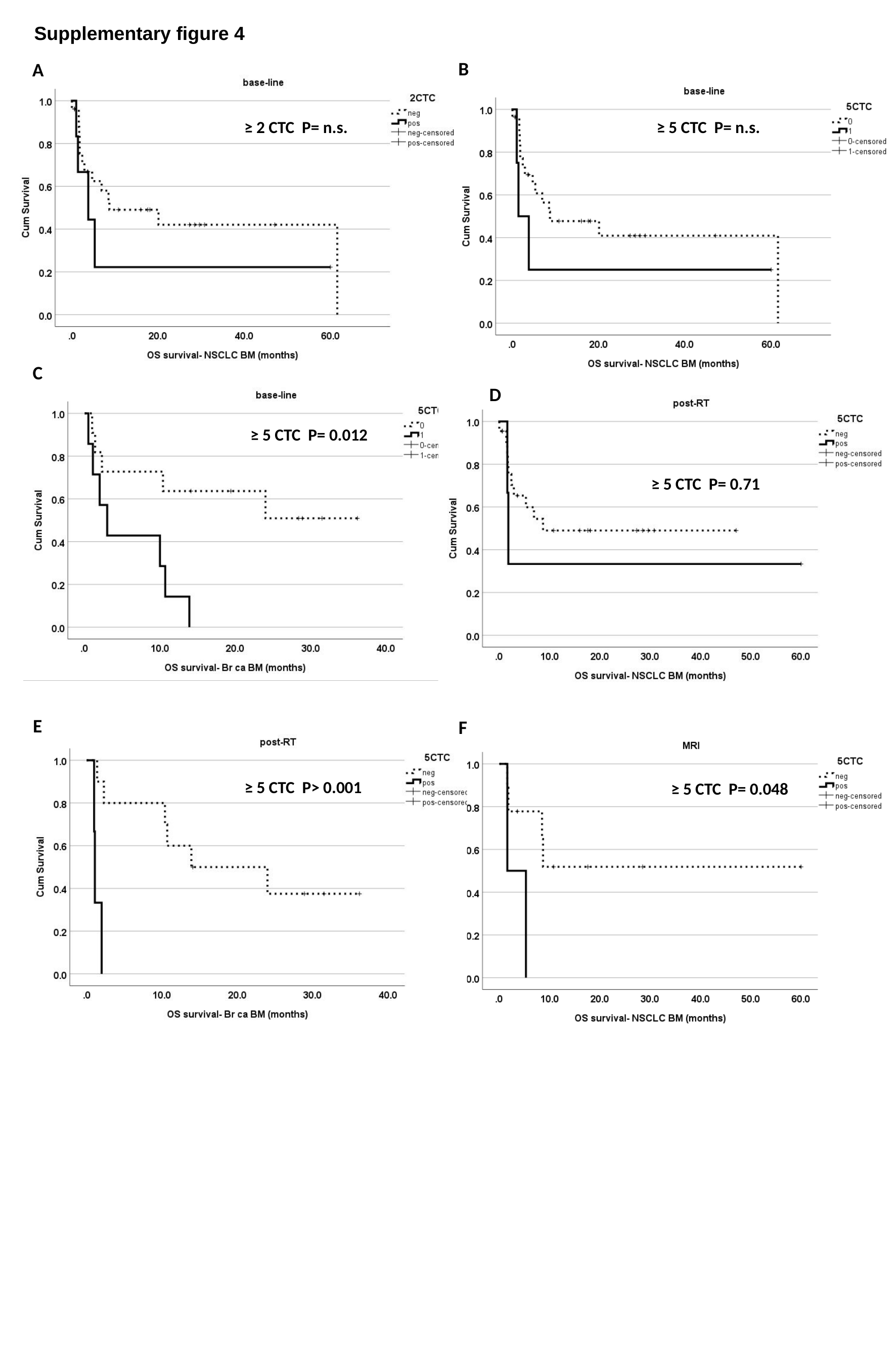

Supplementary figure 4
B
A
≥ 2 CTC P= n.s.
≥ 5 CTC P= n.s.
C
D
≥ 5 CTC P= 0.012
≥ 5 CTC P= 0.71
E
F
≥ 5 CTC P> 0.001
≥ 5 CTC P= 0.048

## Slide 5
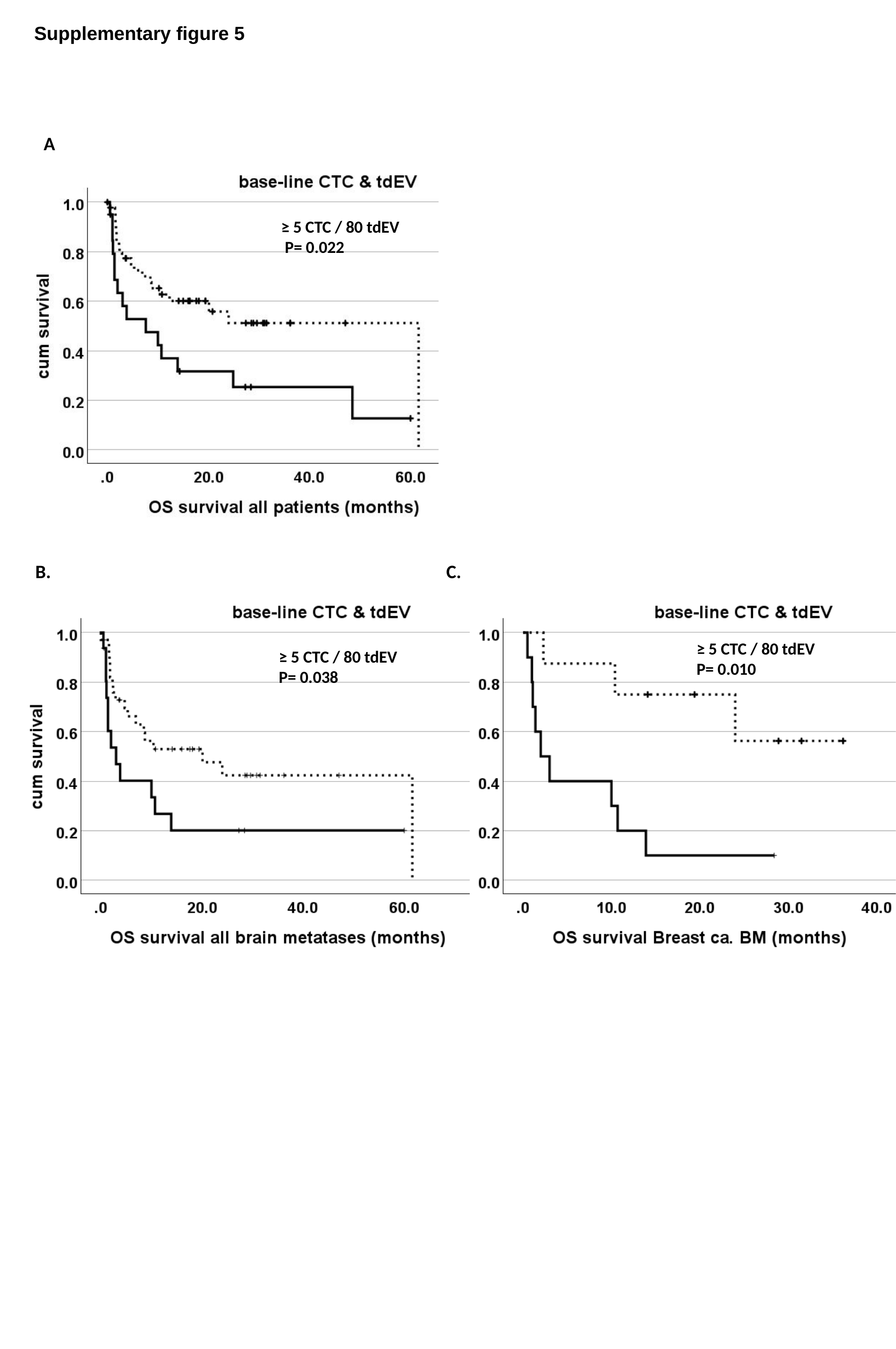

Supplementary figure 5
A
≥ 5 CTC / 80 tdEV
 P= 0.022
B.
C.
≥ 5 CTC / 80 tdEV
P= 0.010
≥ 5 CTC / 80 tdEV
P= 0.038
